# Tectorigenin monohydrate: an isoflavone from *Belamcanda chinensis*

**DOI:** 10.1107/S1600536808030833

**Published:** 2008-10-04

**Authors:** Benguo Liu, Yuxiang Ma, Han Gao, Qiong Wu

**Affiliations:** a School of Food Science, Henan Institute of Science and Technology, Xinxiang 453003, People’s Republic of China; b College of Grain and Food, Henan University of Technology, Zhengzhou 450052, People’s Republic of China; c Jilin Key Laboratory for Biotechnology of Agricultural Products Processing, Changchun University, Changchun 130022, People’s Republic of China

## Abstract

The title compound [systematic name: 5,7-dihydr­oxy-3-(4-hydroxy­phen­yl)-6-meth­oxy-4*H*-chromen-4-one monohydrate], C_16_H_12_O_6_·H_2_O, is isolated from *Belamcanda chinensis* and is said to have anti­microbiotic and anti-inflammatory effects. The  chromen-4-one system and the benzene ring are inclined at a dihedral angle of 36.79 (6)°. Molecules are linked by inter- and intramolecular O—H⋯O hydrogen bonds.

## Related literature

For general background, see: Oh *et al.* (2001 ▶). For a related structure, see: Gao *et al.* (2008 ▶).
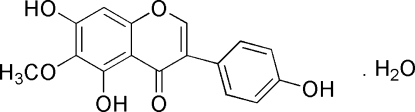


## Experimental

### 

#### Crystal data

C_16_H_12_O_6_·H_2_O
*M* *_r_* = 318.27
Monoclinic, 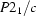

*a* = 12.971 (3) Å
*b* = 14.652 (3) Å
*c* = 7.2930 (15) Å
β = 103.81 (3)°
*V* = 1346.0 (5) Å^3^
*Z* = 4
Mo *K*α radiation
μ = 0.13 mm^−1^
*T* = 113 (2) K
0.14 × 0.04 × 0.02 mm

#### Data collection

Rigaku Saturn CCD area-detector diffractometer
Absorption correction: multi-scan (*CrystalClear*; Rigaku/MSC, 2005 ▶) *T*_min_ = 0.973, *T*_max_ = 0.998
9180 measured reflections
2967 independent reflections
2069 reflections with *I* > 2σ(*I*)
*R*_int_ = 0.085

#### Refinement

*R*[*F* ^2^ > 2σ(*F* ^2^)] = 0.044
*wR*(*F* ^2^) = 0.117
*S* = 1.00
2967 reflections
224 parameters
H atoms treated by a mixture of independent and constrained refinement
Δρ_max_ = 0.33 e Å^−3^
Δρ_min_ = −0.27 e Å^−3^

### 

Data collection: *CrystalClear* (Rigaku/MSC, 2005 ▶); cell refinement: *CrystalClear*; data reduction: *CrystalClear*; program(s) used to solve structure: *SHELXS97* (Sheldrick, 2008 ▶); program(s) used to refine structure: *SHELXL97* (Sheldrick, 2008 ▶); molecular graphics: *SHELXTL* (Sheldrick, 2008 ▶); software used to prepare material for publication: *SHELXTL*.

## Supplementary Material

Crystal structure: contains datablocks I, global. DOI: 10.1107/S1600536808030833/bt2795sup1.cif

Structure factors: contains datablocks I. DOI: 10.1107/S1600536808030833/bt2795Isup2.hkl

Additional supplementary materials:  crystallographic information; 3D view; checkCIF report

## Figures and Tables

**Table 1:** Hydrogen-bond geometry (Å, °)

*D*—H⋯*A*	*D*—H	H⋯*A*	*D*⋯*A*	*D*—H⋯*A*
O7—H7*B*⋯O2^i^	0.77 (3)	2.57 (2)	2.971 (2)	114 (2)
O7—H7*A*⋯O6^ii^	0.95 (3)	1.95 (3)	2.884 (2)	167 (2)
O6—H6⋯O1^iii^	0.88 (2)	1.88 (2)	2.7368 (17)	167 (2)
O3—H3⋯O5	0.90 (2)	1.71 (2)	2.5658 (16)	159.6 (18)
O1—H1⋯O7^iv^	0.87 (2)	1.83 (2)	2.6630 (17)	160.4 (19)

Symmetry codes: (i) 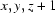; (ii) 
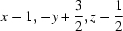
; (iii) 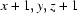; (iv) 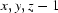.

**Fig. 1.**
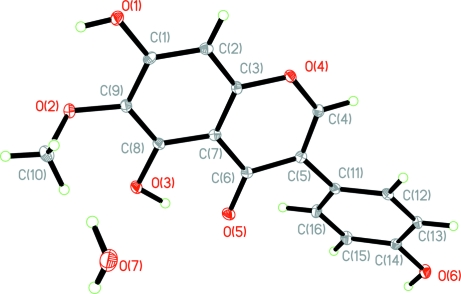
A view of the title compound. Displacement ellopsoids are drawn at the 30% probability level and H atoms are shown as small spheres of arbitrary radii.

## supplementary crystallographic information

### Comment

The title compound [systematic name: 5,7-dihydroxy-3-(4-hydroxyphenyl)-6-methoxy-4*H*-chromen-4-one] was isolated from Belamcanda chinensis and is said to have   antimicrobiotic and anti-inflammatory effects. We report here the crystal structure of its monohydrate. The   two aromatic ring systems rings are inclined at a dihedral angle of 36.79 (6)°. The molecules are linked by intermolecular O—H···O hydrogen bonds (Table 1).

### Experimental

The title compound was isolated from Belamcanda chinensis.

### Refinement

H atoms bonded to C were positioned geometrically (C—H=0.95–0.98 Å), and refined as riding with *U*_iso_(H)=1.2*U*_eq_(C) or 1.5_eq_(C_methyl_). The coordinates of the H atoms bonded to O were refined with *U*_iso_(H)=1.5*U*_eq_(O).

### Crystal data

**Table:**

C_16_H_12_O_6_·H_2_O	*F*(000) = 664
*M**_r_* = 318.27	*D*_x_ = 1.571 Mg m^−^^3^
Monoclinic, *P*2_1_/*c*	Mo *K*α radiation, λ = 0.71073 Å
Hall symbol: -P 2ybc	Cell parameters from 2562 reflections
*a* = 12.971 (3) Å	θ = 1.6–27.1°
*b* = 14.652 (3) Å	µ = 0.13 mm^−^^1^
*c* = 7.2930 (15) Å	*T* = 113 K
β = 103.81 (3)°	Block, colorless
*V* = 1346.0 (5) Å^3^	0.14 × 0.04 × 0.02 mm
*Z* = 4	

### Data collection

**Table:**

Rigaku Saturn CCD area-detector diffractometer	2967 independent reflections
Radiation source: rotating anode	2069 reflections with *I* > 2σ(*I*)
confocal	*R*_int_ = 0.085
Detector resolution: 7.31 pixels mm^-1^	θ_max_ = 27.1°, θ_min_ = 1.6°
ω and φ scans	*h* = −16→16
Absorption correction: multi-scan (CrystalClear; Rigaku/MSC, 2005)	*k* = −15→18
*T*_min_ = 0.973, *T*_max_ = 0.998	*l* = −9→7
9180 measured reflections	

### Refinement

**Table:**

Refinement on *F*^2^	Primary atom site location: structure-invariant direct methods
Least-squares matrix: full	Secondary atom site location: difference Fourier map
*R*[*F*^2^ > 2σ(*F*^2^)] = 0.045	Hydrogen site location: inferred from neighbouring sites
*wR*(*F*^2^) = 0.117	H atoms treated by a mixture of independent and constrained refinement
*S* = 1.00	*w* = 1/[σ^2^(*F*_o_^2^) + (0.0629*P*)^2^] where *P* = (*F*_o_^2^ + 2*F*_c_^2^)/3
2967 reflections	(Δ/σ)_max_ = 0.001
224 parameters	Δρ_max_ = 0.33 e Å^−^^3^
0 restraints	Δρ_min_ = −0.27 e Å^−^^3^

### Special details

**Table:**

Geometry. All e.s.d.'s (except the e.s.d. in the dihedral angle between two l.s. planes) are estimated using the full covariance matrix. The cell e.s.d.'s are taken into account individually in the estimation of e.s.d.'s in distances, angles and torsion angles; correlations between e.s.d.'s in cell parameters are only used when they are defined by crystal symmetry. An approximate (isotropic) treatment of cell e.s.d.'s is used for estimating e.s.d.'s involving l.s. planes.
Refinement. Refinement of *F*^2^ against ALL reflections. The weighted *R*-factor *wR* and goodness of fit *S* are based on *F*^2^, conventional *R*-factors *R* are based on *F*, with *F* set to zero for negative *F*^2^. The threshold expression of *F*^2^ > σ(*F*^2^) is used only for calculating *R*-factors(gt) *etc*. and is not relevant to the choice of reflections for refinement. *R*-factors based on *F*^2^ are statistically about twice as large as those based on *F*, and *R*- factors based on ALL data will be even larger.

### Fractional atomic coordinates and isotropic or equivalent isotropic displacement parameters (Å^2^)

**Table:**

	*x*	*y*	*z*	*U*_iso_*/*U*_eq_	
O1	0.15492 (10)	0.72865 (7)	−0.00354 (17)	0.0204 (3)	
H1	0.1297 (17)	0.6740 (14)	−0.033 (3)	0.031*	
O2	0.26095 (9)	0.56579 (7)	0.09198 (16)	0.0191 (3)	
O3	0.45733 (10)	0.56828 (7)	0.34259 (17)	0.0193 (3)	
H3	0.5174 (18)	0.5889 (13)	0.420 (3)	0.029*	
O4	0.45077 (9)	0.89559 (7)	0.32487 (17)	0.0173 (3)	
O5	0.61065 (9)	0.66388 (7)	0.53713 (16)	0.0188 (3)	
O6	1.02531 (10)	0.87836 (8)	0.95714 (18)	0.0221 (3)	
H6	1.0680 (18)	0.8325 (15)	0.952 (3)	0.033*	
C1	0.25505 (13)	0.72879 (10)	0.1088 (2)	0.0159 (4)	
C2	0.30232 (13)	0.81198 (9)	0.1645 (2)	0.0161 (4)	
H2	0.2660	0.8676	0.1254	0.019*	
C3	0.40402 (13)	0.81206 (9)	0.2788 (2)	0.0142 (4)	
C4	0.55224 (13)	0.89830 (10)	0.4301 (2)	0.0157 (4)	
H4	0.5850	0.9566	0.4520	0.019*	
C5	0.61050 (13)	0.82575 (10)	0.5063 (2)	0.0141 (4)	
C6	0.56447 (13)	0.73485 (10)	0.4688 (2)	0.0142 (4)	
C7	0.45876 (13)	0.73227 (10)	0.3443 (2)	0.0141 (4)	
C8	0.40834 (13)	0.64784 (9)	0.2855 (2)	0.0148 (4)	
C9	0.30772 (14)	0.64647 (9)	0.1659 (2)	0.0155 (4)	
C10	0.22428 (15)	0.50987 (11)	0.2257 (3)	0.0243 (5)	
H10A	0.1689	0.5425	0.2702	0.036*	
H10B	0.2838	0.4963	0.3332	0.036*	
H10C	0.1952	0.4527	0.1648	0.036*	
C11	0.71993 (13)	0.83905 (9)	0.6228 (2)	0.0153 (4)	
C12	0.74392 (14)	0.91262 (9)	0.7482 (2)	0.0157 (4)	
H12	0.6895	0.9541	0.7595	0.019*	
C13	0.84645 (14)	0.92549 (9)	0.8561 (2)	0.0172 (4)	
H13	0.8622	0.9759	0.9401	0.021*	
C14	0.92533 (13)	0.86514 (10)	0.8412 (2)	0.0165 (4)	
C15	0.90422 (14)	0.79282 (10)	0.7145 (2)	0.0197 (4)	
H15	0.9594	0.7526	0.7010	0.024*	
C16	0.80091 (14)	0.78018 (10)	0.6076 (2)	0.0185 (4)	
H16	0.7857	0.7302	0.5225	0.022*	
O7	0.04211 (12)	0.58284 (9)	0.8508 (2)	0.0333 (4)	
H7A	0.033 (2)	0.5862 (15)	0.718 (4)	0.050*	
H7B	0.076 (2)	0.5390 (18)	0.876 (4)	0.050*	

### Atomic displacement parameters (Å^2^)

**Table:**

	*U*^11^	*U*^22^	*U*^33^	*U*^12^	*U*^13^	*U*^23^
O1	0.0144 (7)	0.0182 (6)	0.0251 (7)	0.0000 (4)	−0.0024 (5)	−0.0017 (4)
O2	0.0203 (7)	0.0157 (5)	0.0204 (6)	−0.0030 (4)	0.0031 (5)	−0.0024 (4)
O3	0.0178 (7)	0.0122 (5)	0.0247 (7)	0.0012 (4)	−0.0012 (5)	0.0003 (4)
O4	0.0142 (6)	0.0122 (5)	0.0233 (6)	0.0001 (4)	0.0001 (5)	0.0003 (4)
O5	0.0170 (6)	0.0151 (5)	0.0228 (6)	0.0026 (4)	0.0018 (5)	0.0022 (4)
O6	0.0142 (7)	0.0193 (6)	0.0287 (7)	0.0001 (5)	−0.0033 (5)	−0.0036 (4)
C1	0.0118 (8)	0.0211 (8)	0.0142 (8)	0.0007 (6)	0.0023 (7)	−0.0008 (5)
C2	0.0150 (9)	0.0167 (7)	0.0167 (8)	0.0031 (6)	0.0039 (7)	0.0011 (5)
C3	0.0144 (8)	0.0137 (7)	0.0156 (8)	−0.0020 (6)	0.0060 (7)	−0.0013 (5)
C4	0.0135 (8)	0.0167 (7)	0.0169 (8)	−0.0019 (6)	0.0038 (7)	−0.0018 (5)
C5	0.0129 (8)	0.0162 (7)	0.0144 (8)	0.0003 (6)	0.0054 (7)	−0.0015 (5)
C6	0.0142 (9)	0.0158 (7)	0.0135 (8)	0.0009 (6)	0.0052 (7)	0.0000 (5)
C7	0.0118 (8)	0.0161 (7)	0.0149 (8)	0.0013 (6)	0.0043 (7)	0.0006 (5)
C8	0.0165 (9)	0.0141 (7)	0.0146 (8)	0.0008 (6)	0.0053 (7)	0.0006 (5)
C9	0.0182 (9)	0.0141 (7)	0.0149 (8)	−0.0021 (6)	0.0054 (7)	−0.0012 (5)
C10	0.0250 (10)	0.0205 (8)	0.0285 (10)	−0.0043 (7)	0.0090 (8)	0.0021 (6)
C11	0.0152 (9)	0.0140 (7)	0.0159 (8)	−0.0019 (6)	0.0023 (7)	0.0016 (5)
C12	0.0162 (9)	0.0136 (7)	0.0181 (8)	0.0019 (6)	0.0056 (7)	0.0015 (5)
C13	0.0199 (10)	0.0125 (7)	0.0186 (8)	−0.0029 (6)	0.0033 (7)	−0.0018 (5)
C14	0.0132 (8)	0.0169 (7)	0.0181 (8)	−0.0018 (6)	0.0013 (7)	0.0022 (5)
C15	0.0153 (9)	0.0183 (7)	0.0253 (9)	0.0030 (6)	0.0045 (7)	−0.0031 (6)
C16	0.0177 (9)	0.0179 (7)	0.0192 (9)	−0.0006 (6)	0.0030 (7)	−0.0047 (5)
O7	0.0329 (9)	0.0253 (6)	0.0358 (9)	0.0005 (6)	−0.0037 (7)	−0.0060 (5)

### Geometric parameters (Å, °)

**Table:**

O1—C1	1.3602 (18)	C5—C11	1.483 (2)
O1—H1	0.87 (2)	C6—C7	1.453 (2)
O2—C9	1.3784 (16)	C7—C8	1.4171 (19)
O2—C10	1.439 (2)	C8—C9	1.386 (2)
O3—C8	1.3453 (17)	C10—H10A	0.9800
O3—H3	0.90 (2)	C10—H10B	0.9800
O4—C4	1.3568 (18)	C10—H10C	0.9800
O4—C3	1.3717 (16)	C11—C16	1.384 (2)
O5—C6	1.2434 (17)	C11—C12	1.400 (2)
O6—C14	1.3823 (18)	C12—C13	1.387 (2)
O6—H6	0.88 (2)	C12—H12	0.9500
C1—C2	1.381 (2)	C13—C14	1.376 (2)
C1—C9	1.400 (2)	C13—H13	0.9500
C2—C3	1.382 (2)	C14—C15	1.390 (2)
C2—H2	0.9500	C15—C16	1.393 (2)
C3—C7	1.3917 (19)	C15—H15	0.9500
C4—C5	1.345 (2)	C16—H16	0.9500
C4—H4	0.9500	O7—H7A	0.95 (3)
C5—C6	1.458 (2)	O7—H7B	0.77 (3)
			
C1—O1—H1	113.5 (13)	O2—C9—C8	121.26 (13)
C9—O2—C10	114.16 (13)	O2—C9—C1	118.99 (13)
C8—O3—H3	100.2 (12)	C8—C9—C1	119.62 (13)
C4—O4—C3	118.50 (11)	O2—C10—H10A	109.5
C14—O6—H6	112.1 (13)	O2—C10—H10B	109.5
O1—C1—C2	118.13 (13)	H10A—C10—H10B	109.5
O1—C1—C9	120.40 (13)	O2—C10—H10C	109.5
C2—C1—C9	121.47 (13)	H10A—C10—H10C	109.5
C1—C2—C3	118.09 (13)	H10B—C10—H10C	109.5
C1—C2—H2	121.0	C16—C11—C12	118.55 (14)
C3—C2—H2	121.0	C16—C11—C5	120.86 (13)
O4—C3—C2	116.81 (12)	C12—C11—C5	120.58 (15)
O4—C3—C7	120.39 (13)	C13—C12—C11	120.59 (15)
C2—C3—C7	122.80 (13)	C13—C12—H12	119.7
C5—C4—O4	125.74 (13)	C11—C12—H12	119.7
C5—C4—H4	117.1	C14—C13—C12	119.88 (14)
O4—C4—H4	117.1	C14—C13—H13	120.1
C4—C5—C6	118.67 (14)	C12—C13—H13	120.1
C4—C5—C11	119.95 (13)	C13—C14—O6	117.93 (14)
C6—C5—C11	121.36 (13)	C13—C14—C15	120.68 (14)
O5—C6—C7	121.41 (13)	O6—C14—C15	121.39 (15)
O5—C6—C5	123.52 (14)	C14—C15—C16	118.98 (16)
C7—C6—C5	115.08 (12)	C14—C15—H15	120.5
C3—C7—C8	117.94 (13)	C16—C15—H15	120.5
C3—C7—C6	121.37 (13)	C11—C16—C15	121.29 (14)
C8—C7—C6	120.69 (13)	C11—C16—H16	119.4
O3—C8—C9	119.11 (12)	C15—C16—H16	119.4
O3—C8—C7	120.86 (13)	H7A—O7—H7B	102 (2)
C9—C8—C7	120.03 (13)		
			
O1—C1—C2—C3	179.92 (17)	C10—O2—C9—C8	73.9 (2)
C9—C1—C2—C3	0.2 (3)	C10—O2—C9—C1	−110.33 (18)
C4—O4—C3—C2	−177.22 (16)	O3—C8—C9—O2	−5.6 (3)
C4—O4—C3—C7	2.1 (3)	C7—C8—C9—O2	173.72 (17)
C1—C2—C3—O4	177.11 (17)	O3—C8—C9—C1	178.63 (17)
C1—C2—C3—C7	−2.2 (3)	C7—C8—C9—C1	−2.1 (3)
C3—O4—C4—C5	−4.9 (3)	O1—C1—C9—O2	6.3 (3)
O4—C4—C5—C6	2.7 (3)	C2—C1—C9—O2	−173.97 (17)
O4—C4—C5—C11	−178.42 (17)	O1—C1—C9—C8	−177.84 (17)
C4—C5—C6—O5	−177.81 (18)	C2—C1—C9—C8	1.9 (3)
C11—C5—C6—O5	3.3 (3)	C4—C5—C11—C16	−139.51 (19)
C4—C5—C6—C7	2.0 (3)	C6—C5—C11—C16	39.4 (3)
C11—C5—C6—C7	−176.86 (16)	C4—C5—C11—C12	39.3 (3)
O4—C3—C7—C8	−177.26 (17)	C6—C5—C11—C12	−141.85 (18)
C2—C3—C7—C8	2.0 (3)	C16—C11—C12—C13	−0.7 (3)
O4—C3—C7—C6	2.6 (3)	C5—C11—C12—C13	−179.49 (16)
C2—C3—C7—C6	−178.17 (18)	C11—C12—C13—C14	−0.5 (3)
O5—C6—C7—C3	175.31 (19)	C12—C13—C14—O6	−177.31 (16)
C5—C6—C7—C3	−4.5 (3)	C12—C13—C14—C15	2.1 (3)
O5—C6—C7—C8	−4.8 (3)	C13—C14—C15—C16	−2.4 (3)
C5—C6—C7—C8	175.30 (17)	O6—C14—C15—C16	176.94 (16)
C3—C7—C8—O3	179.49 (17)	C12—C11—C16—C15	0.3 (3)
C6—C7—C8—O3	−0.4 (3)	C5—C11—C16—C15	179.12 (17)
C3—C7—C8—C9	0.2 (3)	C14—C15—C16—C11	1.2 (3)
C6—C7—C8—C9	−179.67 (17)		

### Hydrogen-bond geometry (Å, °)

**Table:**

*D*—H···*A*	*D*—H	H···*A*	*D*···*A*	*D*—H···*A*
O7—H7B···O2^i^	0.77 (3)	2.57 (2)	2.971 (2)	114 (2)
O7—H7A···O6^ii^	0.95 (3)	1.95 (3)	2.884 (2)	167 (2)
O6—H6···O1^iii^	0.88 (2)	1.88 (2)	2.7368 (17)	167 (2)
O3—H3···O5	0.90 (2)	1.71 (2)	2.5658 (16)	159.6 (18)
O1—H1···O7^iv^	0.87 (2)	1.83 (2)	2.6630 (17)	160.4 (19)

Symmetry codes: (i) *x*, *y*, *z*+1; (ii) *x*−1, −*y*+3/2, *z*−1/2; (iii) *x*+1, *y*, *z*+1; (iv) *x*, *y*, *z*−1.
